# Population structure and transmission modes of indigenous typhoid in Taiwan

**DOI:** 10.1186/s12920-019-0576-6

**Published:** 2019-09-03

**Authors:** Kai-Yu Wang, De-Jen Lee, Shian-Sen Shie, Chih-Jung Chen

**Affiliations:** 1 School of medicine, College of Medicine, Chang Gung University, 333 Taoyuan, Taiwan; 2 Physical Education Office, Chang Gung University, 333 Taoyuan, Taiwan; 3 Division of Infectious Diseases, Department of Internal Medicine, Chang Gung Memorial Hospital, 333 Taoyuan, Taiwan; 4 Division of Pediatric Infectious Diseases, Department of Paediatrics, Chang Gung Memorial Hospital, Linkou, No. 5, Fu-Shin Street, Kweishan, 333 Taoyuan, Taiwan; 5 Department of Pediatrics, Xiamen Chang Gung Hospital, Xiamen, Fujian China

**Keywords:** Typhoid fever, Whole-genome sequencing, Pulsed-field gel electrophoresis, Transmission mode, Taiwan

## Abstract

**Background:**

Indigenous typhoid fever was continuing to be identified in Taiwan which has not been endemic for the enteric fever for more than 20 years. The source and transmission by which the local patients acquired typhoid and the population structure of the indigenous typhoid strains remain not well characterized.

**Methods:**

During 2001 and 2014, non-duplicated clinical *Salmonella enterica* serovar Typhi isolates in a hospital were analyzed by whole-genome sequencing (WGS) and determined for pulsotypes. Maximum likelihood phylogeny was constructed by nucleotide alterations in core genomes and compared to the framework of global typhoid strains. Potential source and transmission were traced by correlating the phylogeny and the temporal relationship between isolates.

**Results:**

A total of 43 S. Typhi isolates from indigenous cases were analyzed and a majority (39, 90.7%) of them were belonged to six WGS-defined genotypes prevailing mainly in Southeast Asia. Genotype 3.4.0 and a multidrug-resistant type 4.3.1 (also known as pandemic H58 haplotype) were associated respectively with two solitary small-scale outbreaks, implying a transmission mode of importation followed by outbreak. Twelve isolates with nearly identical core genomes were belonged to genotype 3.2.1 but were categorized into three different pulsotypes. The 3.2.1 isolates were identified across 13 years and involved in three clusters and a sporadic case, indicating sustained local transmission of the same strain. The remaining indigenous isolates belonging to three genotypes (2.1, 3.1.2, and 3.0.0) were of substantial genetic diversity and isolated at different time points, indicating independent event of each case.

**Conclusions:**

Indigenous typhoid in Taiwan occurred mainly with the forms of small-scale outbreaks or sporadic events likely by contracting imported strains which prevailed in Southeast Asia. Sustained local transmission of certain strain was also evident by WGS analysis, but not by conventional pulsotyping, highlighting the importance of continuing molecular surveillance of typhoid fever with adequate tools in the non-endemic region.

**Electronic supplementary material:**

The online version of this article (10.1186/s12920-019-0576-6) contains supplementary material, which is available to authorized users.

## Background

Typhoid fever is a multisystemic illness caused by *Salmonella enterica* serovar Typhi (*S*. Typhi). Human is the only natural host and drinking water and food are the main vehicle for transmission of S. *Typhi* [1, 2]. The infected cases can transmit bacteriae to other people at their acute illness and 1 to 5% of the cases recovered from typhoid fever become carriers [3]. The chronic carriers were often the cause of epidemics of typhoid. The disease burden remained high in low- and middle-income countries in central Africa, Central, South, and Southeast Asia [4, 5].

Similar to the condition in most of the developed countries, the incidence of typhoid fever in Taiwan was very low and cases were sporadically reported in the past two decades. A significant proportion of the reported cases were imported from the endemic regions. Nevertheless, the indigenous cases were not uncommon and account for 330 (58.1%) of 568 reported cases during 2001 and 2014 according to Taiwan Centers of Disease Control [6]. It remained not completely understood where the sources could be and by which modes the transmission occurred among the indigenous cases. *S.* Typhi is a genetically monomorphic pathogen and tracing its phylogeny has been challenging. Pulsed-field gel electrophoresis (PFGE) was considered the golden standard for epidemiological surveillance and outbreaks investigation of a variety of Salmonella species. Unfortunately, PFGE was also described as unreliable, subjective method and hard to communicate between laboratories [7]. In the last decade, single nucleotide polymorphism (SNP)-based typing methods have successfully established phylogenetic markers for discriminating *S.* Typhi subtypes [8, 9]. However, the discrimination power remains not satisfactory. Inspired by the recent research identifying phylogenetically informative clades of global *S.* Typhi strains [10], the study was aimed to clarify the phylogenetic relatedness among the indigenous strains and between the indigenous and global strains by whole genome sequencing (WGS). The genomic structures and phylogenetic information of strains based on the WGS data allowed the identification of potential geographic source of the indigenous cases. A variety of phenotypes of the *S.* Typhi strains, including the antibiotic resistance and virulence, can also potentially be explored by the WGS method [11, 12]. More importantly, by combing the temporal and genetic information, we were able to recognize the potential transmission modes by which the typhoid took place in the community in this island.

## Methods

### Strains

The *S.* Typhi isolates used in the study were all clinical strains identified during 2001 and 2014 in Chang Gung Memorial Hospital (CGMH). The strains were identified by standard methods. The BD BACTEC™ FX blood culture system (BD Diagnostics, Sparks, MD) were used for the blood samples. Positive samples were plated onto 5% sheep BAP/EMB agar, chocolate agar and AN-BAP agar at 37 °C for 8–10 h. *Salmonella* was identified by matrix-assisted laser desorption ionization–time of flight mass spectrometry (MALDI-TOF MS, Bruker). *S.* Typhi was confirmed using biochemical tests followed by serogrouping using slide agglutination with positive results of polyvalent A – I & Vi, group D and Vi.

CGMH is the only medical center located in suburban region in northern Taiwan and provided primary to tertiary care to patients of all ages. An estimate of three million population resided in this region. During 2001 and 2014, a total of 60 clinical *S.* Typhi isolates were available in the strain bank of CGMH. After reviewing the medical records, information of travel history was not available in two Taiwanese patients and the two responsible isolates were excluded from the analysis. The remaining 58 isolates were categorized into two groups respectively of imported and indigenous isolates. An imported isolate was defined if (1) an isolate recovered from patients within 90 days of their arrival at or returning to Taiwan or (2) an isolate from a foreigner patient.

### Whole genome sequencing (WGS)

The genomic DNA was extracted using Tissue & Cell Genomic DNA Purification Kit (GeneMark, Taiwan). The Nextera XT DNA Library Preparation Kit (Illumina, CA, USA) was used for constructing the sequencing library with 0.2 ng of total input genomic DNA. The average fragment size was 500 bp. The sequencing was performed with Illumina MiSeq System (Illumina, San Diego CA, USA) using 2X300bp paired-end protocol to obtain 200X coverage. A range of 87.9 to 169.6-fold base coverage across the genomes was achieved by dividing the total bases output with the genome length. De novo assembly was conducted using SPAdes 3.11.1 [13]. This Whole Genome Shotgun project has been deposited at DDBJ/ENA/GenBank under accession number PRJNA437172. Scaffold outputs greater than 200 bp were used in the following analysis.

To determine the phylogenetic relatedness of the indigenous isolates and the global isolates, the short read raw data of global strains of the interested genotypes were downloaded from the Sequence Read Archive using the fast-dump toolkit. The assembly of draft genome of each isolate was also conducted with the same SPAdes 3.11.1 procedure [13].

### Phylogenetic analysis

The multi-alignment of the core genomes was conducted with the parsnp script [14]. *S.* Typhi strain CT18 was used as the reference genome during the procedure. The multi-alignment file was subsequently used for maximum likelihood phylogeny construction based on the SNPs outside of the potential recombination regions with the gubbins procedure [15]. The final phylogenetic tree was input into Interactive Tree Of Life (http://itol.embl.de) for further annotation.

### Determination of the genotypes, harbored resistance genes and plasmids

The WGS-defined genotyping was conducted in accordance with the typing system of S. Typhi proposed by Wong et al. [10]. The detection of resistance genes and plasmids were conducted with the srst2 script using the database of ARG-ANNOT, and PlasmidFinder, respectively [16, 17].

### Pulse-field gel electrophoresis (PFGE)

PFGE was performed in accordance with the procedure described elsewhere [18]. The plug with bacteriae was digested with restriction enzyme *XbaI* and PFGE was carried out with a CHEF Mapper XA System (Bio-Rad Laboratories). BIONUMERICS software (Applied Maths, Kortrijk, Belgium) was used to analyze the DNA fingerprints produced by PFGE.

### Susceptibility

The *S*. Typhi isolates were determined for susceptibilities to a variety of antimicrobial agents including ampicillin, chloramphenicol, cefixime, ciprofloxacin, ceftriaxone, flomoxef, imipenem and trimethoprim-sulfamethoxazole. Susceptibilities to all antimicrobial agents except flomoxef were determined using the standard disc diffusion method according to the CLSI 2016 (M100-S27) guideline, and susceptibility to flomoxef depends on the standard given by drug manufacture.

## Results

### Demographics and origins of Typhi isolates

**Fig. 1:**
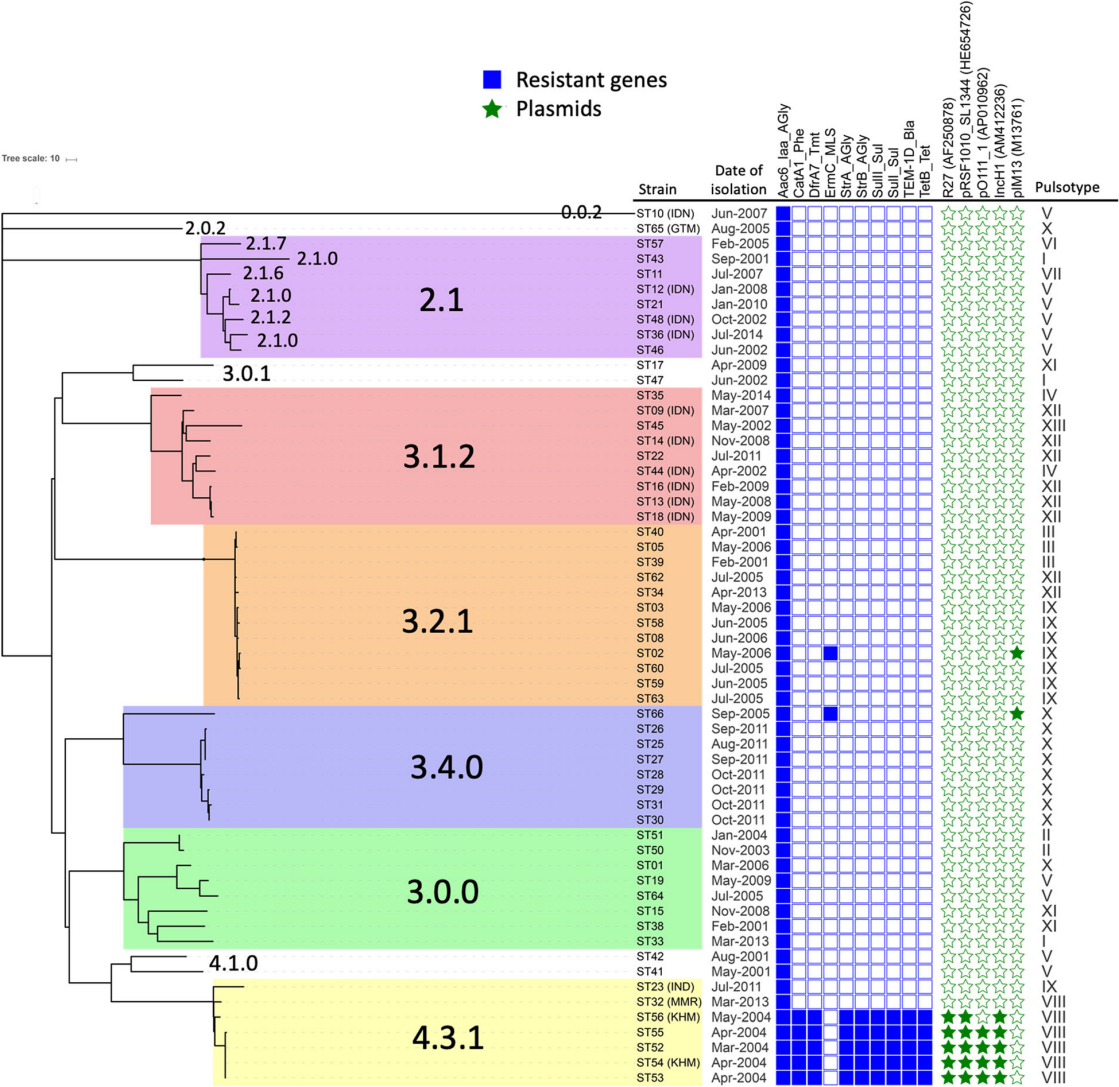
Whole genome-based phylogenetic tree, clades, resistome and carried plasmids in 58 S. Typhi isolates in Taiwan. Isolates of six major clades were highlighted by six different color. The strain names followed by parentheses indicate imported strains and the exported countries are labelled by three capital letters within parentheses. The blue square indicates presence of the resistant genes and the green star indicates presence of the indicated plasmids. Abbreviation: GTM, Guatemala; IDN, Indonesia; IND, India; KHM, Cambodia; MMR, Myanmar

Among the 58 *S.* Typhi isolates, 43 (74.1%) were isolated from indigenous cases and 15 (25.9%) were imported from endemic countries including Indonesia (10 isolates), Cambodia (2 isolates), India (1 isolate), Myanmar (1isolate) and Guatemala (1 isolate). A total of 10 genotypes including six major types were identified in the 58 isolates according to the proposed WGS-defined typing system [10]. The six major types were clade 2.1 (8 isolates), clade 3.1.2 (9 isolates), clade 3.2.1 (12 isolates), clade 3.4.0 (8 isolates), clade 3.0.0 (8 isolates) and clade 4.3.1 (7 isolates). The detailed origin of isolates, core-genome based phylogenetic relatedness, WGS-defined-genotypes, PFGE types and the differentially carried resistant genes are shown in Fig. 1 and Additional file 1: Figure S1.

### Genomic features of Typhi isolates

Screening of the 58 whole genomes of the *S.* Typhi strains identified 10 resistant genes in chromosome or plasmids mediating resistance to seven classes of antimicrobial agents (Fig. 1). All of the isolates carried an Aac6_Iaa gene in the chromosome mediating resistance to aminoglycoside. *ErmC* gene on the plasmid pIM13 conferring macrolide-lincosamide-streptogramin B resistance phenotype was found in two isolates, ST02 and ST60, respectively of clade 3.2.1 and 3.4.0 [19]. The remaining eight resistant genes were exclusively harbored in the IncH1 plasmid by five isolates of clade 4.3.1. Clade 4.3.1 was a multi-drug resistant (MDR) clone also known as H58 pandemic clone [20, 21]. Consistent with the resistome, the five 4.3.1 isolates exhibited multi-resistance to ampicillin, chloramphenicol and trimethoprim-sulfamethoxazole to which the strains of other genotypes and two imported strains (ST23 and ST32) of clade 4.3.1 absent for IncH1 remained susceptible (Table 1).

**Table 1:** Antibiotics susceptibilities in 58 *Salmonella* Typhi isolates in northern Taiwan, 2001–2014

Antibiotic	Susceptibility, N (%)			*P* value
	All isolates *n* = 58	Isolates of clade 4.3.1 *n* = 7	Isolates not of clade 4.3.1 *n* = 51	
Ampicillin	47 (81.0)	2 (28.6)	49 (96.1)	< 0.001
Chloramphenicol	47 (81.0)	2 (28.6)	49 (96.1)	< 0.001
Cefixime	58 (100)	7 (100)	51 (100)	…
Ciprofloxacin	57 (98.3)	6 (85.7)	51 (100)	0.121
Ceftriaxone	58 (100)	7 (100)	51 (100)	…
Flomoxef	58 (100)	7 (100)	51 (100)	…
Imipenem	58 (100)	7 (100)	51 (100)	…
TMP-SXT	47 (81.0)	2 (28.6)	49 (96.1)	< 0.001

Abbreviation: TMP-SXT, Trimethoprim-Sulfamethoxazole

### Potential transmission modes of typhoid

#### Imported and outbreak

**Fig. 2:**
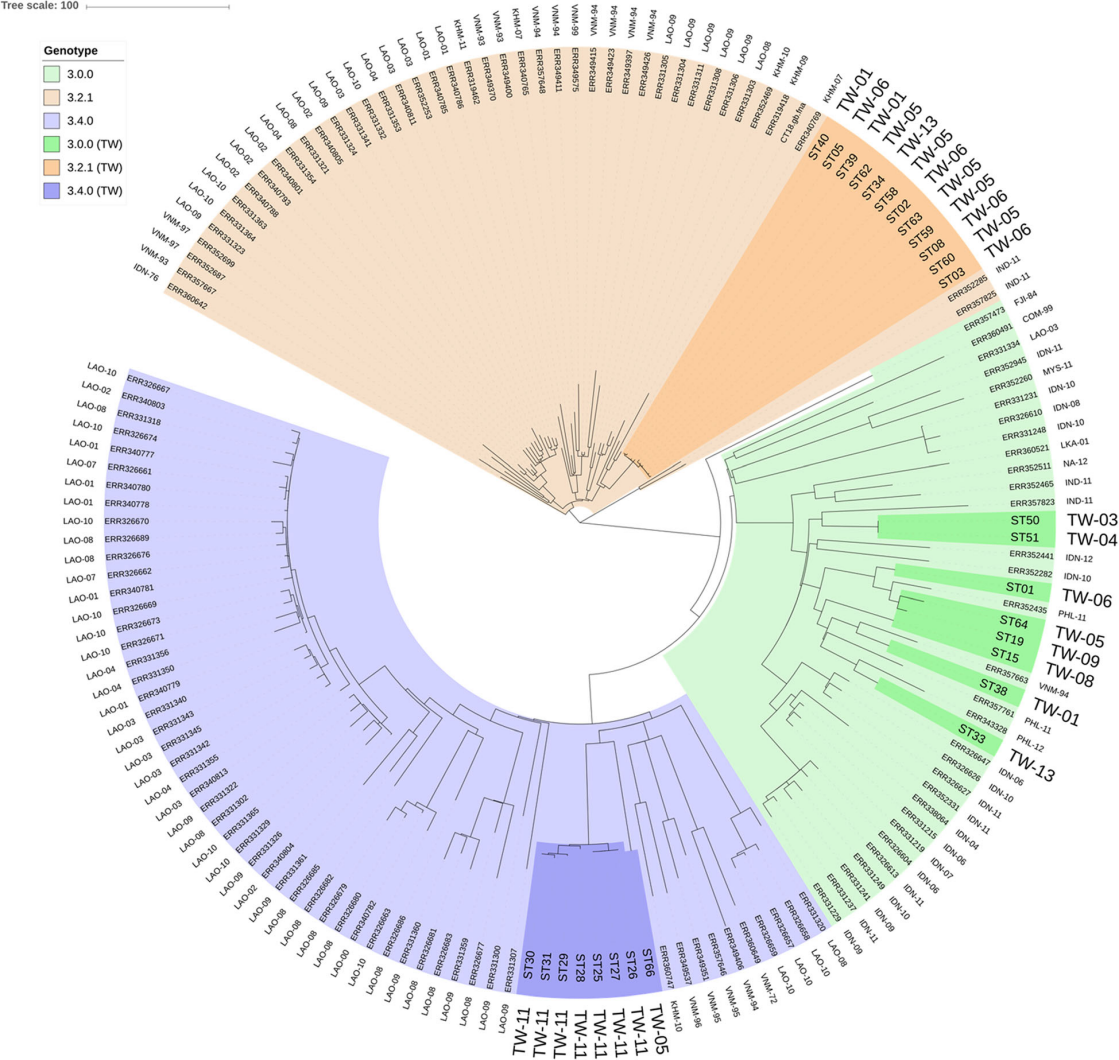
Phylogeographical clustering of S. Typhi strains of three major clades isolated from Taiwan and the strains of the same clades in global population framework. The strains highlighted by dark color were indigenous isolates from Taiwan. The names of the countries from which the strains were isolated and the years of isolation were labelled at outer ring. Abbreviation: LAO, Lao; KHM, Cambodia; VNM, Vietnam; IDN, Indonesia; PHL, Philippines; IND, India; NA, Namibia; LKA, Sri Lanka; MYS, Malaysia; COM, Colombia; FJI, Fiji; TW, Taiwan

Except for isolate ST66, the indigenous strains of clade 3.4.0 were identified exclusively in three consecutive months (August to October) in 2011 and were of very close genetic relatedness with a maximal number of 18 SNPs in the core genomes (between ST26 and ST31) (Fig. 1). The global strains of genotype 3.4.0 were most commonly reported in Laos and Vietnam. Comparison of the 3.4.0 strains from Taiwan, Vietnam, Laos and Cambodia disclosed that the Taiwanese strains were within a solitary clade (Fig. 2). The observations strongly suggested local transmission of the 3.4.0 strains in Taiwan. It was likely that the strain was imported from endemic areas to Taiwan in 2011 and caused a typhoid outbreak involving at least 7 patients in this region. The outbreak appeared to be well controlled as no further 3.4.0 strain was identified in the following years.

Strains of MDR clone 4.3.1 was another example of ‘Imported and outbreak’ transmission mode. We found that there were three 4.3.1 indigenous isolates (ST52, ST53, ST55) identified in March and April 2004 in this cohort, which were very close related to an isolate imported from Cambodia (ST54), with identical core genomes in isolate ST52, ST53 and ST54 and only one SNP in isolate ST55 comparing to other three isolates (Fig. 1). Further, the four strains harbored the same profiles of resistome and plasmids as mentioned above. The data indicated that the 4.3.1 strain imported from Cambodia caused an outbreak in March and April of 2004 in Taiwan. Similar to the condition of 3.4.0 strains, the cluster of 4.3.1 strains in 2004 was successfully controlled and no further case was identified afterward.

#### Repeated importation and limited transmission

Strains of genotype 3.0.0 in this cohort was identified sporadically in different years and across almost the entire period of the study from February 2001 to March 2013. Except for the phylogenetic related strains ST50 and ST51, the 3.0.0 strains were of wide genomic diversity with 102 to 148 nucleotides difference in their core genomes at pairwise comparisons. The data suggested that the 3.0.0 strains were repeatedly imported into Taiwan and transmitted limitedly to few individuals. The transmission was not sustained as only one strain was identified in each year in this cohort. The speculation was supported by the phylogeny data of the international 3.0.0 strains, which disclosed that at least three geographic sources could be traced for the Taiwanese strains (Fig. 2). Strains ST01, ST15, ST19, ST33 and ST64 were related to the strains from Philippines, strain ST15 and ST38 were related to a strain from Vietnam and strains ST50 and ST51 were related to a strain isolated in India. The data strongly suggested that the 3.0.0 *S*. Typhi isolates in Taiwan did not propagate from a single origin but transmitted by repeated importation.

A majority of the strains of clades 3.1.2 and 2.1 and minor genotypes including 3.0.1 and 4.1.0 were also belonged to this ‘repeated importation and limited transmission’ mode of *S.* Typhi infections. Even though with the same genotype, the indigenous strains were of relative distant phylogenetic relatedness and isolated sporadically in different years, indicating different source of the strains (Fig. 1). The small number of cases further suggested the limited transmission of these strains after their importation into a non-endemic region.

#### Sustained local transmission

Strains of genotype 3.2.1 was an example of sustained local transmission in non-endemic regions. Among the 12 indigenous isolates, the first isolate was identified as early as in April 2001 and the last one was isolated in April 2013. They were all very close related strain as the number of nucleotide difference in core genomes was between 1 and 9 in pairwise comparisons (Fig. 1). Three outbreaks caused by this strain occurred in 2001, 2005 and 2006 respectively involved 2, 5 cases and 4 cases respectively. It appeared that the locally circulating 3.2.1 strain in Taiwan was related to the strains from Cambodia (Fig. 2).

## Discussion

Results from the current study demonstrated the continuing occurrences of indigenous typhoid fever with the form of either sporadic events or outbreaks in Taiwan during the past two decades. By the cutting-edge technology of WGS, we were able to comprehend the population structure of the *S.* Typhi isolates circulating in this island. More importantly, we were for the first time able to precisely delineate the phylogenetic relatedness between strains and the transmission modes through which the *S.* Typhi isolates were spreading in this non-endemic area. The data regarding how the indigenous typhoid cases occurred in the community was critical information for disease control. Indeed, the WGS analysis clearly disclosed that half of the indigenous typhoid cases were belonging to different outbreaks involving three major clades 3.2.1, 3.4.0 and 4.3.1 (Fig. 1). The outbreaks caused by the strain of clade 3.2.1 were of particular public health importance because the single strain was circulating at this region across at least 13 years and associated with at least three outbreaks. Unfortunately, it appeared that the source was never identified and the strain was continuing contaminating the environment. Without the WGS analysis, it would be very difficult to link the 3.2.1 strains-associated outbreaks and sporadic infections, as the responsible isolates might have been categorized into several different genotypes according to the PFGE typing method (Fig. 1 and Additional file 1: Figure S1).

Indeed, by PFGE typing the 58 *S.* Typhi isolates in the current study could be categorized into a total of 13 major pulsotypes when the cutoff similarity of band pattern was set at 75% (Fig. 1 and Additional file 1: Figure S1). Unexpectedly, we found poor correlations between the PFGE types and the WGS-based genotypes for a substantial proportion of strains. Of note, a total of three PFGE types (III, IX and XII) can be identified in the 12 strains of clade 3.2.1, though the WGS phylogeny of the strains indicated a very closed genetic relatedness. It was very likely that the typhoid cases infected by the same 3.2.1 strain had been classified as distinct clusters caused by three different strains according to the PFGE typing. In another aspect, some phylogenetically diverse isolates in WGS-based analysis were grouped into the same PFGE type. For instance, the PFGE type I was assigned to isolates ST33, ST43 and ST47, but were respectively categorized into three different WGS-defined clades, 2.1.0, 3.0.1 and 3.0.0 (Fig. 1). The data combined together demonstrated the unreliability of the PFGE typing system in defining the genetic relatedness among S. Typhi strains. The advantage of WGS-based genotyping over the once considered gold standard method for typing *S.* Typhi isolates was obvious. The WGS should be considered in replacement of the enzyme digestion methods in the epidemiological surveillance and outbreak investigations of salmonella infections including typhoid fever [22].

In the current study, three major WGS-defined clade (2.1, 3.1.2 and 4.3.1) were shared by indigenous strains and imported strains from Southeastern Asia especially Indonesia. A highly-resistant strain carrying the IncH1 plasmid with multiple resistant genes imported from Cambodia (ST54) was even identical to indigenous isolates of genotype 4.3.1, indicating direct transmission between the imported and indigenous typhoid cases. Intriguingly, the multi-resistance was also identified in another strain from Cambodia but not in two strains imported from India and Myanmar. Further analysis of the global strains disclosed two groups of the 4.3.1 strains respectively with or without the IncH1 plasmid carrying multiple drug resistant genes (Additional file 2: Figure S2). The carriage of resistant genes in strains from different countries may result from a variety of environmental and host factors including use of antimicrobial agents, dietary habits and gut microbiota of the residences. The observation suggested the imported cases from Southeast Asia were the main source of indigenous typhoid in Taiwan. Timely identification of the potential source with easily implemented, affordable and accurate diagnostic methods followed by adequate infection control measure will be the critical step to interrupt the transmission of bacteriae and reduce the burden of typhoid fever in the non-endemic areas [23].

## Conclusions

In conclusion, the *S.* Typhi strains isolated from Taiwanese population without abroad travel history were mostly belonged to six major clades including a pandemic clade (H58 haplotype) with plasmid-mediated multidrug resistance prevailing in Southeast Asia. Importation followed by small-scale outbreaks and repeated importation from the endemic region with limited local circulations were two common transmission modes of indigenous typhoid cases in this island. Further, sustained transmissions of a single strain across more than a decade were identified and might not be readily recognized by the traditional enzyme digestion typing methods. Our finding highlighted the importance of continuing epidemiologic surveillance of the enteric fever in non-endemic regions by adequate genotyping tools such as WGS. The geographic sources of the local strains can also be easily and accurately predicted at the country or region levels by comparison of the WGS data to the global population framework.

## Additional files

Additional file 1: **Figure S1.** The PFGE patters of 58 S. Typhi isolates and the year of isolation in Taiwan, 2001–2014 (TIF 924 kb)

Additional file 2: **Figure S2.** Phylogenetic tree, carried resistant genes and plasmids of international S. Typhi isolates of a multiresistant clade 4.3.1. The red squares indicate the presence of resistant genes and the blue stars indicate presence of the plasmids. The countries from which the strains were isolated are labelled with three capital letters. Abbreviation: BGD, Bangladesh; KEN, Kenya; IND, India; LKA, Sri Lanka; MMR, Myanmar; PAK, Pakistan; VNM, Vietnam; KHM, Cambodia; TWN, Taiwan; LAO, Lao. (TIF 1437 kb)

## Data Availability

The Whole Genome Shotgun project has been deposited at DDBJ/ENA/GenBank under accession number PRJNA437172.

## Abbreviations

CGMH: Chang Gung Memorial Hospital
CLSI: Clinical & Laboratory Standard Institute
MDR: multi-drug resistant
PFGE: Pulsed-field gel electrophoresis
*S.* Typhi: *Salmonella enterica* serovar Typhi
SNP: single nucleotide polymorphism
WGS: whole-genome sequencing
