# Supplementary figures and images for: Population structure and transmission modes of indigenous typhoid in Taiwan
Source: BMC Med Genomics. 2019 Sep 3;12:126. doi: 10.1186/s12920-019-0576-6 (PMC6724314; doi:10.1186/s12920-019-0576-6)

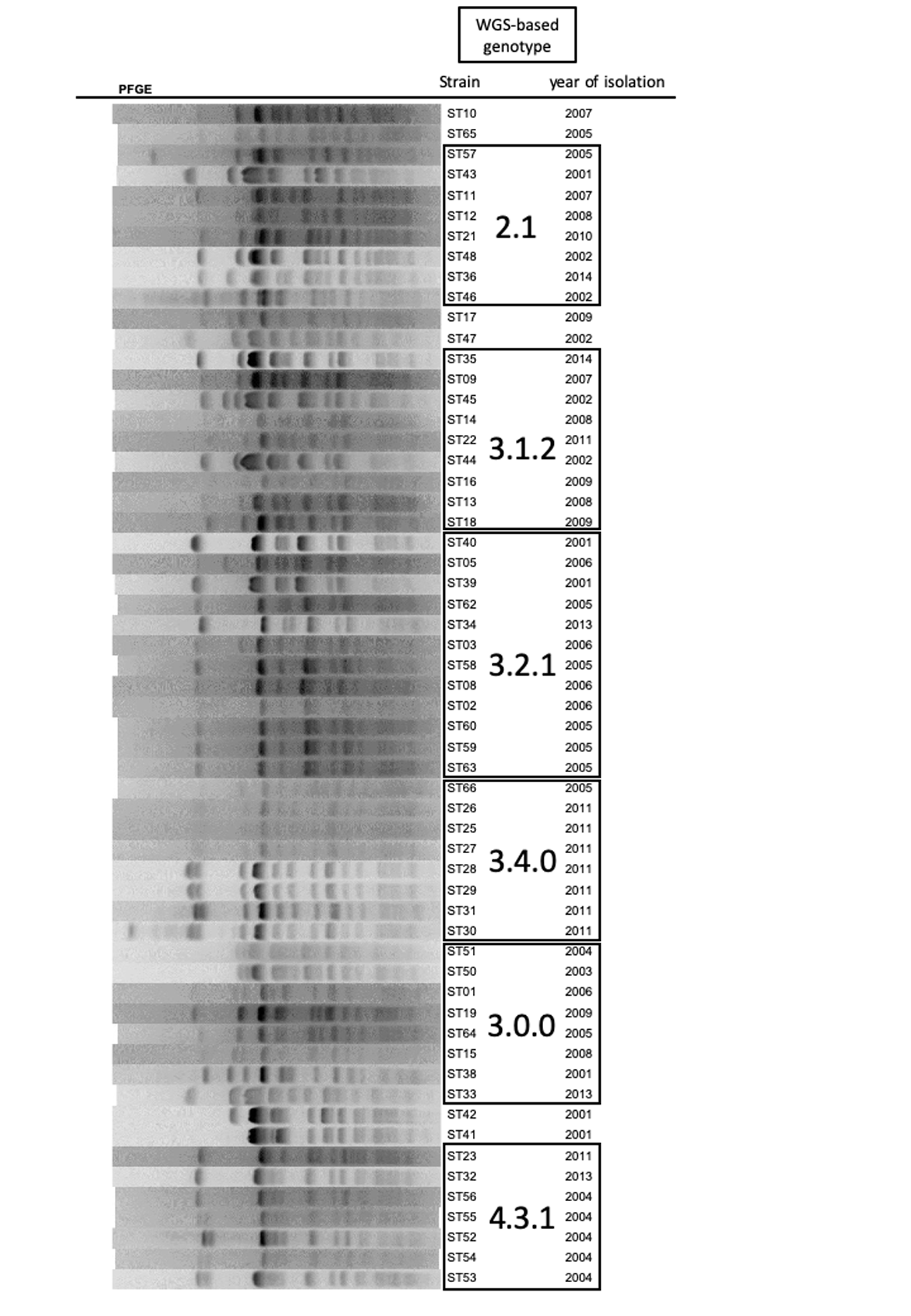

Supplement: Supplementary file 1 — Figure S1. The PFGE patters of 58 S. Typhi isolates and the year of isolation in Taiwan, 2001–2014 (TIF 924 kb) [file 12920_2019_576_MOESM1_ESM.tif]

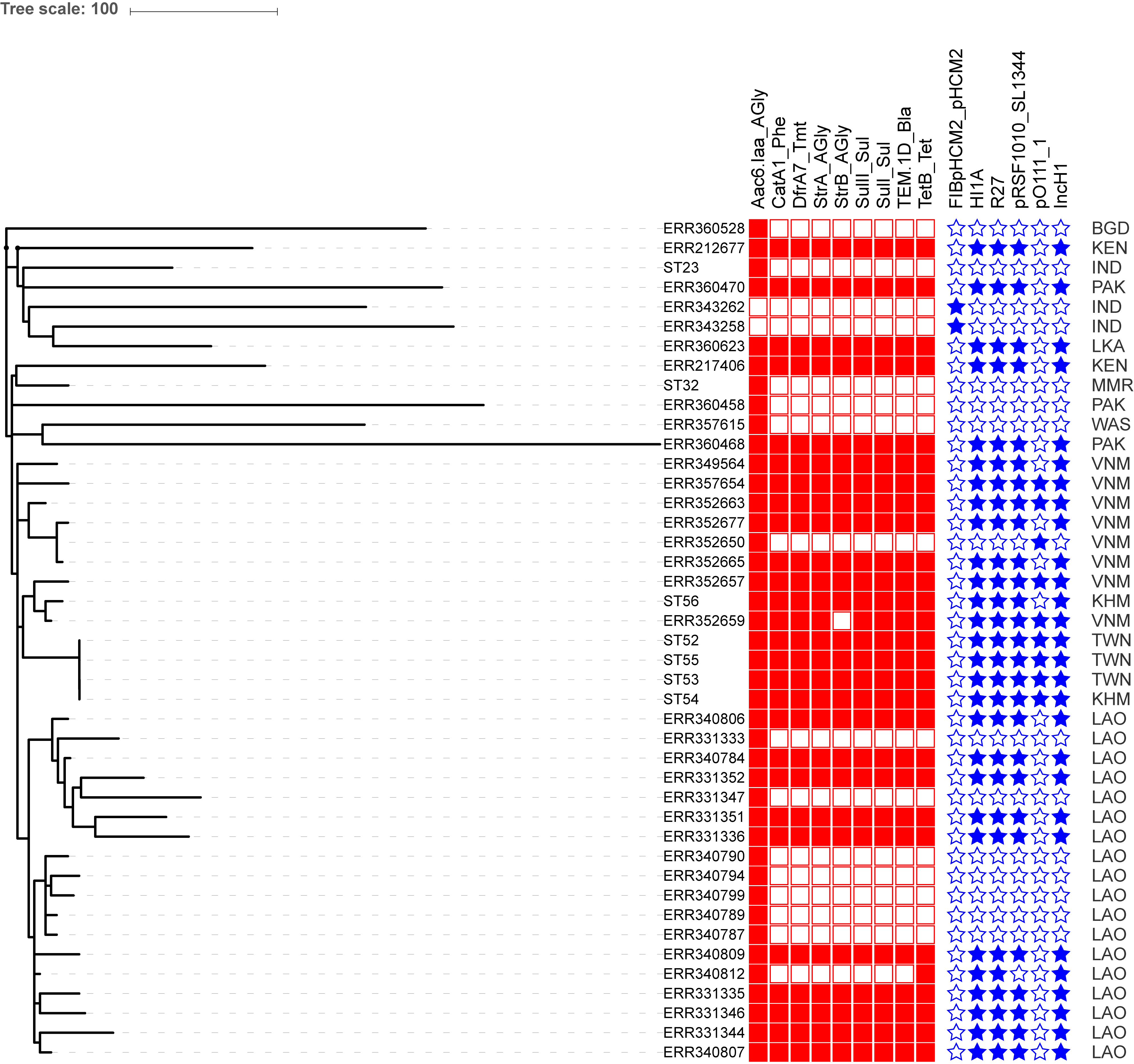

Supplement: Supplementary file 2 — Figure S2. Phylogenetic tree, carried resistant genes and plasmids of international S. Typhi isolates of a multiresistant clade 4.3.1. The red squares indicate the presence of resistant genes and the blue stars indicate presence of the plasmids. The countries from which the strains were isolated are labelled with three capital letters. Abbreviation: BGD, Bangladesh; KEN, Kenya; IND, India; LKA, Sri Lanka; MMR, Myanmar; PAK, Pakistan; VNM, Vietnam; KHM, Cambodia; TWN, Taiwan; LAO, Lao. (TIF 1437 kb) [file 12920_2019_576_MOESM2_ESM.tif]
